# Treatment Strategies for Patients with Mitral Regurgitation: A Meta-Analysis of Randomized Controlled Trials

**DOI:** 10.3390/jpm15080383

**Published:** 2025-08-16

**Authors:** Claudia Carassia, Fiorenzo Simonetti, Hector A. Alvarez Covarrubias, Bernhard Wolf, Costanza Pellegrini, Tobias Rheude, Patrick Fuchs, Ferdinand Roski, Moritz Kühlein, Edna Blum, Gjin Ndrepepa, Teresa Trenkwalder, Michael Joner, Adnan Kastrati, Salvatore Cassese, Erion Xhepa

**Affiliations:** 1 Cardiology Department, Busto Arsizio Hospital, ASST Valle Olona, 21052 Busto Arsizio, Italy; claudia.carassia@asst-valleolona.it; 2 Klinik für Herz- und Kreislauferkrankungen, TUM Klinkum Deutsches Herzzentrum, Technische Universität München, 80636 Munich, Germanycassese@dhm.mhn.de (S.C.); xhepa@dhm.mhn.de (E.X.); 3 Department of Advanced Biomedical Sciences, University of Naples Federico II, 80131 Naples, Italy; 4 Departamento de Cardiología, Hospital de Cardiología, Centro Médico Nacional Siglo XXI, Instituto Mexicano del Seguro Social (IMSS), Mexico City 06720, Mexico; 5 DZHK (German Center for Cardiovascular Research), Partner Site Munich Heart Alliance, 80336 Munich, Germany

**Keywords:** degenerative mitral regurgitation, functional mitral regurgitation, meta-analysis, personalized medicine, surgical mitral valve repair, transcatheter mitral valve repair

## Abstract

**Background**: Several treatment strategies are available for patients with mitral valve regurgitation (MR). However, evidence regarding their comparative effectiveness remains limited. We sought to compare the performance of different treatment strategies for personalized treatment of patients with MR. **Methods**: We performed a pairwise and network meta-analyses of randomized trials comparing treatment strategies for patients with MR. Patients were divided in two groups: transcatheter mitral valve repair (TMVR, including edge-to-edge repair and indirect percutaneous annuloplasty) and control (surgery or optimal medical therapy). The primary outcome of this analysis was all-cause death. Main secondary outcomes were re-hospitalization for heart failure and re-intervention. **Results**: A total of seven trials with 2324 participants, with mainly functional MR (TMVR, *n* = 1373-control, *n* = 951) were available for the quantitative synthesis. The median follow-up duration was 14 months. Compared to control therapy, TMVR significantly reduced all-cause death (RR 0.77, 95% CI 0.65–0.91, *p* = 0.002) and re-hospitalization for heart failure (RR 0.67, 95% CI 0.49–0.91, *p* = 0.01). Among TMVR strategies, the edge-to-edge repair with MitraClip ranked as possibly the best option to reduce all-cause death. **Conclusions**: In symptomatic patients with significant MR, TMVR is associated with a significant reduction of all-cause death, and re-hospitalization for heart failure, mainly in patients with functional MR. Additional comparative studies are needed to investigate the best TMVR treatment option, for patients with degenerative MR.

## 1. Introduction

Mitral regurgitation (MR) is one of the most prevalent valvular heart diseases on a global scale, with a significant impact on mortality, morbidity, and quality of life [1,2]. Although mitral valve surgery has been the only available non-pharmacological treatment for MR for several decades, only a minority of patients with significant MR ultimately undergo corrective surgery due to an elevated operative risk [3]. This unmet need has fueled intensive research directed towards the development of alternative, personalized, less invasive therapies, which could potentially widen the treatment strategies over high surgical risk.

In the last years, several transcatheter mitral valve repair (TMVR) strategies have become available for the treatment of MR. Current TMVR strategies can be broadly classified into transcatheter edge-to-edge repair (TEER, MitraClip [*Abbott Vascular*] and Pascal [*Edwards Lifesciences*] systems), relying on the concept of leaflet approximation, and direct and indirect mitral valve annuloplasty (Carillon, *Cardiac Dimensions*).

Despite the broadening portfolio of transcatheter treatment options, the available evidence that assesses their efficacy and safety, and their comparative effectiveness with surgical and medical treatment, remains controversial. Additionally, the underlying mechanism of MR is of paramount importance, since degenerative (DMR) and functional MR (FMR) display major differences in terms of natural history and clinical outcomes following both surgical and transcatheter treatment [4,5,6,7,8]. The widest evidence to date, deriving from large, prospective randomized controlled trials (RCT), pertains to the efficacy and safety of TEER in patients presenting with FMR as compared to optimal medical therapy (OMT) [9,10,11]; moreover, based on the results of RCTs and retrospective and prospective registries, TEER is currently recommended for patients with DMR at high surgical risk [7,8,12,13,14]. However, these trials display significant heterogeneity in terms of devices under investigation and MR aetiology; additionally, they were not powered to evaluate hard clinical endpoints individually. Previous meta-analyses investigating TMVR focused on a specific aetiology or treatment and did not compare TMVR strategies [15,16].

Against this background, we performed a meta-analysis of RCTs evaluating clinical outcomes of TMVR as compared to alternative treatment strategies in patients with MR, and to assess the comparative performance of different TMVR strategies in this setting in order to investigate a possible personalized approach in patients with MR.

## 2. Materials and Methods

**Search strategy.** PubMed, EMBASE, Medline, and Clinical Trials.gov were searched up to September 2024 for studies to be included in the quantitative synthesis using the following keywords: “mitral regurgitation”, “mitral valve repair”, “transcatheter mitral valve repair”, “transcatheter edge-to-edge repair”, “mitral annuloplasty”, “MitraClip”, “Pascal” and “Carillon” (Table S1). We applied the following inclusion criteria: (i) RCTs comparing any type of TMVR either against each other or against surgery or OMT in patients with significant (grade 2+ or more), symptomatic MR (ii) mortality data of any cause available in both treatment arms. We excluded registries, single-arm studies, cohort studies, duplicate publications, as well as articles published in a language other than English. Data from post hoc analyses from RCTs were also excluded.

**Assessment of risk of bias in included studies.** The Cochrane Risk-of-Bias tool for randomized trials (RoB 2) was used to assess trial eligibility. Ratings of bias were categorized into low risk, unclear risk, and high risk. The quality of evidence was extracted by two independent investigators (CC and FS), while disagreement was solved by a third investigator (SC).

**Outcome measures.** The primary outcome of this analysis was all-cause death. The main secondary outcomes were re-hospitalization due to heart failure and mitral valve re-intervention, including intervention for residual MR for patients receiving OMT. Additional secondary outcomes were MR recurrence grade 3+ or 4+, and NYHA class III/IV at follow-up. All outcomes were evaluated within a time frame of 24 months following randomization.

**Statistical analysis.** Firstly, we performed a pairwise meta-analysis, comparing a TMVR strategy versus control treatment (either surgery or OMT). Mean and standard deviations for continuous variables and percentages and frequencies for categorical variables were shown as exploratory analyses for baseline features of participants enrolled in each included study. The weighted median follow-up duration was calculated based on the sample size of each individual study. Risk ratios (RRs) with 95% confidence intervals (95% CI) and *p*-values < 0.05 were used to compare outcomes of interest between treatment groups. Study-level risk estimates were pooled using the Mantel–Haenszel random-effect model [17].

Secondly, we performed a network meta-analysis to generate direct and indirect evidence between interventions according to the definition of intention-to-treat analyses in the original trials. The random-effects model was used to estimate the risk for all-cause death, the main outcome of this analysis. The quality of the network of evidence was assessed by evaluating weights, comparisons, and influence of individual studies for these outcomes. Further details concerning the statistical framework of the pairwise and network meta-analyses are reported in the Supplementary Methods (Supplementary Data).

This study was reported in compliance with the Preferred Reporting Items for Systematic reviews and Meta-Analyses (PRISMA) statement (Table S2) [18]. All analyses were performed using the packages netmeta, meta, and metafor in R (version 4.1.3-R Foundation for Statistical Computing, Vienna, Austria) and with RevMan (Version 5.4. The Cochrane Collaboration, 2020). No extramural funding was used to support this work. Ethical approval was not required for this study. The protocol for this systematic review and meta-analysis was not registered.

## 3. Results

### 3.1. Eligible and Included Studies

A total of 2731 records were identified, whilst 111 records were screened at title level, 30 at abstract level, and 12 were assessed for eligibility—five records were excluded because of being ongoing trials [19,20] or post hoc analysis and extended follow-up of included trials [21,22,23], with seven RCTs finally included in the present meta-analysis (Figure S1) [9,10,11,12,13,24,25]. Patients were divided in two groups according to treatment allocation (TMVR vs. control). A list of trials and endpoints is provided in Table S3. Six trials were included in the pairwise meta-analysis, including a total of 2030 patients. Of these, 1079 patients were assigned to TMVR (MitraClip, *n* = 992-Carillon, *n* = 87) and 951 to control therapy (surgery, *n* = 199-OMT, *n* = 752). The CLASP IID trial [13] was excluded from the pairwise analysis due to direct comparison between two TMVR strategies, but was included in the network meta-analysis. The flow diagram for the trial selection process is shown in Figure S1. The risk of bias assessed using the Rob2 is summarized in Figure S2. Three trials, COAPT, EVEREST, and RESHAPE HF2, displayed a high risk, while the CLASP IID trial resulted in unclear risk. The main characteristics of enrolled patients are shown in Table 1. Of note, five trials analyzed patients with FMR, one trial included patients with DMR, and one trial included primarily patients with DMR. The weighted median follow-up available for this analysis was 14 months.

### 3.2. Primary Outcome

**Figure 1:**
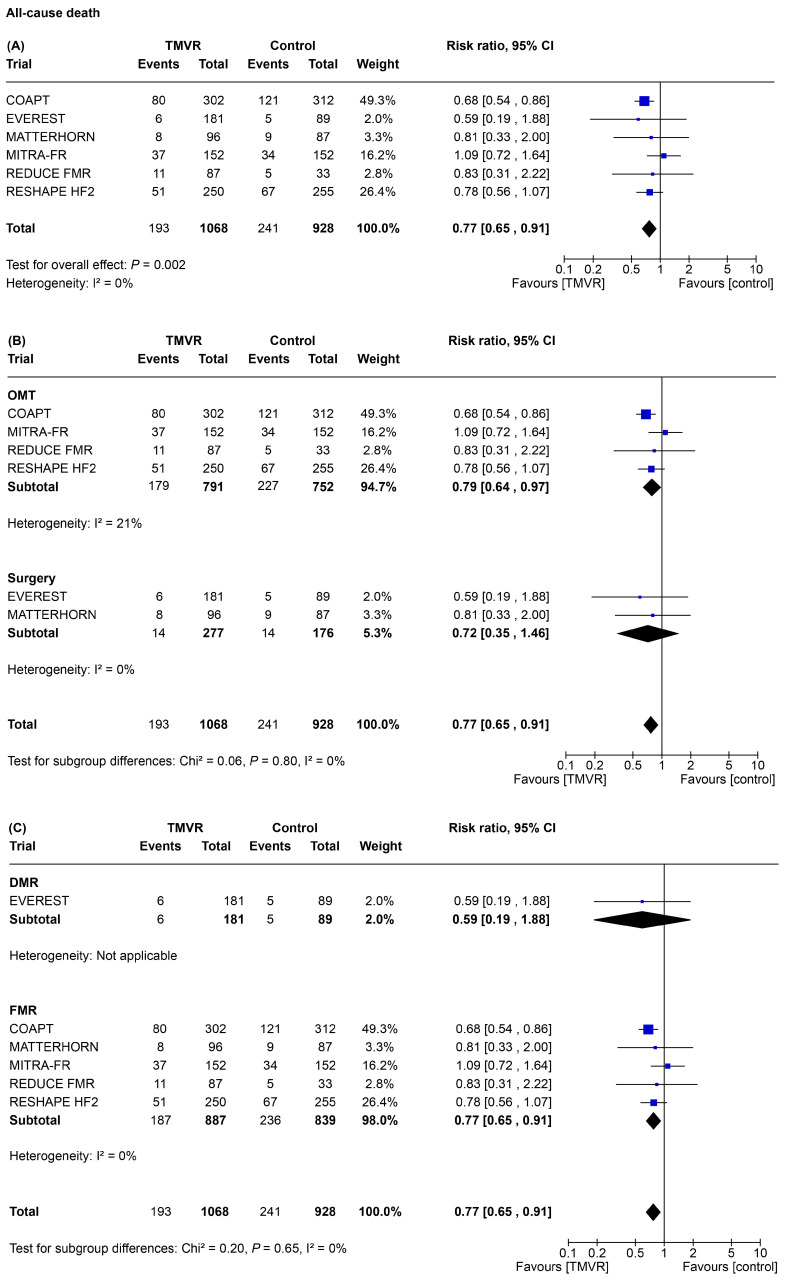
Forest plot of risk of all-cause death. Metanalysis of included studies revealed that in patients with MR, TMVR significantly reduced all-cause death compared to control (**A**) and to OMT alone (**B**). Comparison between TMVR and surgery showed a non-significant reduction in all-cause death. Subgroup analysis for MR etiology (**C**) shows significant reduction in all-cause mortality in patients with FMR, but not in patients with DMR. CI, confidence interval; DMR, degenerative mitral regurgitation; FMR, functional mitral regurgitation; MR, mitral regurgitation; OMT, optimal medical therapy; TMVR, transcatheter mitral valve repair.

All-cause death occurred in 434 patients (22%), of whom 193 (18%) in the TMVR group and 241 (26%) in the control group. Compared to control, TMVR significantly reduced the risk of all-cause death (RR 0.77, 95% CI 0.65–0.91-*p* = 0.002, Figure 1A). The random effects meta-analysis had a statistical power of 93% to detect a 25% relative risk difference for the primary endpoint associated with a TMVR strategy. The subgroup analysis for all-cause death showed a significant treatment effect in favor of TMVR as compared to OMT (RR 0.79, 95% CI 0.64–0.97, *p* = 0.02, Figure 1B) and in patients with FMR (RR 0.77, 95% CI 0.65–0.91, *p* = 0.002, Figure 1C); however, without a significant statistical interaction (Table 2). Of note, in the subgroup of patients with DMR there were few fatal events. The visual inspection of the funnel plot and the linear regression test for funnel plot asymmetry displayed no publication bias for the primary outcome (*p* = 0.66; Figure S3). However, the limited number of trials does not allow to definitively exclude a possible publication bias. The influence analysis for all-cause death displayed consistent results with the main analysis, with overall low heterogeneity (Table S4).

### 3.3. Secondary Outcomes

**Figure 2:**
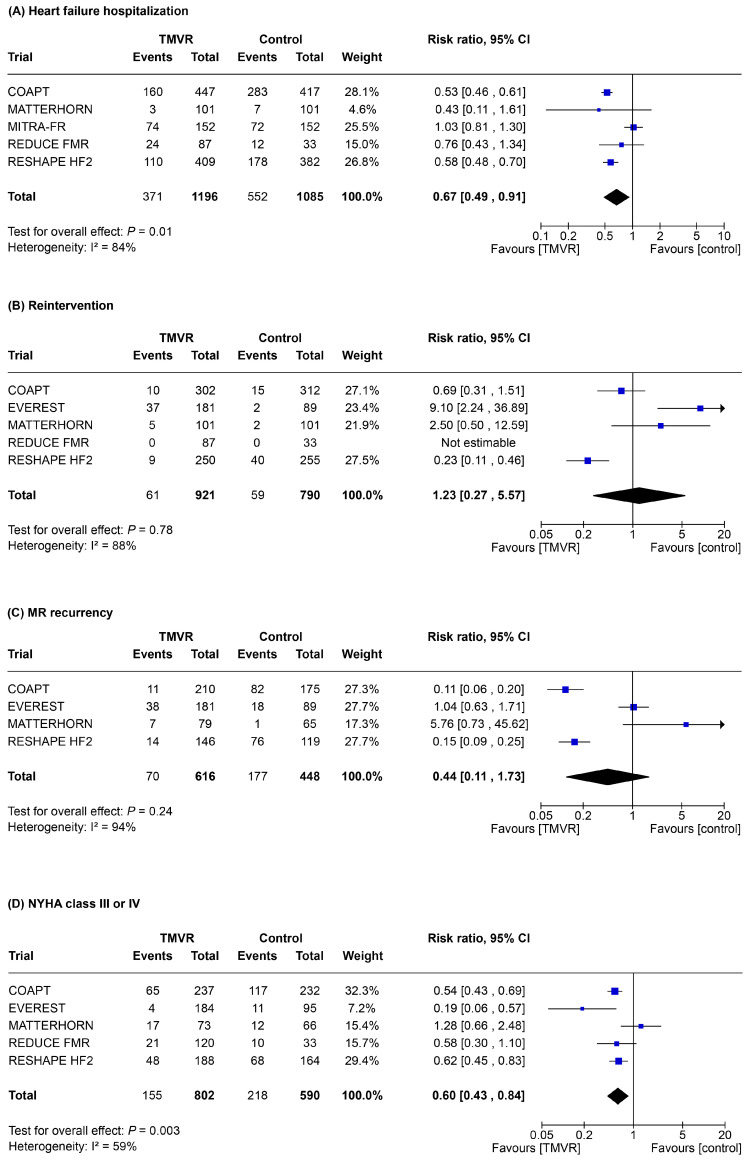
Forest plot of risk of heart failure hospitalization (**A**), re-intervention (**B**), MR recurrency (**C**), and NYHA Class III or IV (**D**). CI, confidence interval; NYHA, New York Heart Association; MR, mitral regurgitation; TMVR, transcatheter mitral valve repair.

Results for secondary outcomes are presented in Figure 2. Re-hospitalization for heart failure occurred in 923 (40%) patients. Compared to control therapy, TMVR significantly reduced re-hospitalization for heart failure (RR 0.67, 95% CI 0.49–0.91; *p* = 0.01; Figure 2A). Mitral valve re-intervention occurred in 120 (7%) patients. There was no significant difference between TMVR and control therapy in terms of re-intervention (RR 1.23, 95% CI 0.27–5.57; *p* = 0.78; Figure 2B). Both these outcomes displayed high statistical heterogeneity.

Compared to control therapy, the reduction of the recurrence of significant (grade 3+ or 4+) MR did not reach statistical significance (RR 0.44, 95% CI 0.11–1.73; *p* = 0.24), while showing a significant improvement of patient functional class (RR 0.60, 95% CI 0.44–0.84, *p* = 0.003; Figure 2C,D).

The results of subgroup analysis for primary and secondary outcomes are reported in Table 2. Importantly, a significant interaction was observed with underlying MR etiology for all secondary outcomes as well as with the type of control therapy in terms of mitral valve re-intervention and recurrence of significant MR. Compared to surgery alone, TMVR showed a trend towards higher rates of MR recurrence as well as significantly higher rates of re-intervention. An explorative analysis excluding trials with a high risk of bias showed results consistent with the main analysis, with loss of statistical significance in some subgroups, related to the larger sample size of trials considered at high risk of bias.

### 3.4. Network Meta-Analysis

The network of treatment strategies for all-cause death, the primary outcome, is reported in Figure S4. Notably, the vast majority of comparisons were indirect in nature. Consequently, the network meta-analysis included 2324 patients (MitraClip *n* = 1087, Pascal *n* = 199, Carillon *n* = 87, surgery *n* = 199, medical therapy *n* = 752).

**Figure 3:**
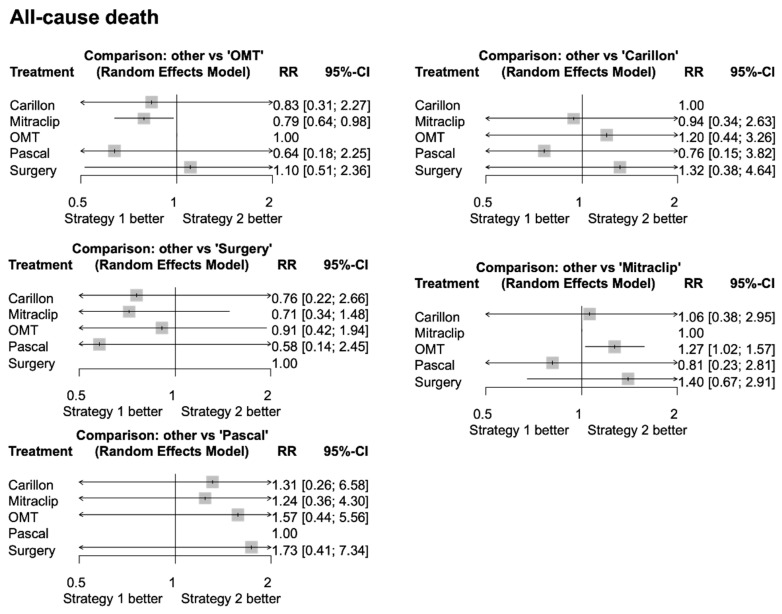
Forest plot from network meta-analysis for all-cause death showing results from direct and indirect comparison between different treatments. The forest plots of pooled risk ratios and 95% CI for all-cause death are derived by network meta-analysis. CI, confidence interval; OMT, optimal medical therapy; RR, risk ratio. Comparable results were found for re-hospitalization for HF (Figures S7 and S8). Compared to OMT, only TEER with MitraClip significantly reduced re-hospitalization for HF (RR 0.67, 95% CI 0.47–0.97; *p* = 0.035).

Compared to OMT, only TEER with MitraClip significantly reduced all-cause death (RR 0.79, 95% CI 0.64–0.98, *p* = 0.03), whereas other TMVR strategies showed a trend towards reduction of all-cause death, with large confidence intervals and lack of statistical significance. Compared to surgery, all percutaneous strategies showed a trend towards reduction of all-cause death, without reaching statistical significance. These results are reported in Figure 3 and Figure S5. TEER with MitraClip ranked as the best option in terms of prevention of all-cause death as compared to other strategies (*p*-score = 0.70), followed by Pascal, Carillon, OMT, and surgery. A direct comparison of the various treatment strategies revealed a symmetrical distribution in the funnel plot, with all studies located outside the area of statistical significance for publication bias, and all corresponding *p*-values consistently above 0.1. Therefore, on the basis of visual inspection (Figure S6) and statistical assessment through Egger’s test (*p* = 0.48), it can be concluded that both a small-study effect and a significant publication bias can be excluded.

## 4. Discussion

In the present meta-analysis, we investigated the efficacy, safety and the comparative effectiveness of available treatment strategies for MR, assessed in the setting of prospective RCTs. The main findings of the present study can be summarized as follows:

(i). In symptomatic patients with significant MR, TMVR was associated with a significant reduction of all-cause death, re-hospitalization for heart failure, MR, and improved patient functional class as compared to control therapy;
(ii). These results are mainly driven by trials enrolling patients with FMR. Due to the limited data available, however, they cannot be extended to patients presenting with DMR. For this subgroup of patients, the results of the present meta-analysis should therefore be considered exploratory in nature;
(iii). TEER appears to be the best treatment for mortality reduction, due to the extent of evidence regarding the MitraClip device;
(iv). Further trials are needed to obtain direct stronger evidence in the context of DMR, and to provide direct comparison between different strategies in order to improve the personalized treatment of patients with MR.

The recent evidence supporting a beneficial effect of TMVR on all-cause mortality has been conflicting, partly related to discordant outcomes of clinical trials [9,10,26]. Additionally, all of the trials investigated mortality as a secondary outcome or as a part of a composite primary outcome, none of them was individually powered to assess differences in all-cause mortality. By pooling the results of available randomized clinical studies, the present meta-analysis achieved sufficient power to draw reliable conclusions regarding mortality, and shows a significant reduction in mortality in patients treated with TMVR as compared to control therapy.

However, the underlying aetiology of MR is of paramount importance, both in terms of natural history and clinical outcomes following surgical or transcatheter treatment. Such differences are reflected in the different classes of recommendation assigned to surgical and transcatheter treatments by both ESC and ACC/AHA guidelines, according to the underlying mitral valve pathology (DMR vs. FMR) [7,8].

Moreover, the amount of evidence provided for these two entities is entirely different: five out of the six RCTs included in the present meta-analysis focus on patients displaying FMR and only one trial investigated patients with DMR [12]. As a consequence, by showing significant improvements in terms of all-cause mortality, HF hospitalizations and virtually all secondary outcomes, the present analysis lends to support a wider adoption of TMVR, mainly in patients with FMR. These findings are primarily driven by the favorable clinical outcomes following TEER, performed by means of the MitraClip device in the large majority of available studies, and are supported by the findings of several large, rigorously conducted, prospective RCTs.

On the other hand, patients displaying DMR represent only a minority of those included in available randomized clinical trials. Consequently, the present meta-analysis reveals no substantial differences between percutaneous and surgical repair in terms of all-cause mortality in patients with DMR. However, TMVR has been associated with a significantly higher incidence of re-intervention. Nevertheless, the present analysis remains descriptive due to the scarcity of evidence and the high statistical heterogeneity. Moreover, the EVEREST II was performed with the first generation MitraClip and its results were reported >10 years ago. In the contemporary era, TMVR operators have accumulated greater experience and TMVR technology has undergone significant refinement, characterized by the emergence of new generations of devices and advanced delivery systems. These developments have enabled the treatment of a more extensive range of mitral valve anatomies, yielding favorable outcomes.

However, the most recent scientific evidence in the context of DMR relied on prospective real-world registries [27,28,29,30,31] and, has demonstrated superior clinical and echocardiographic outcomes following TEER with contemporary device generations. It is noteworthy that not only have mortality rates declined over time and with the adoption of more advanced devices, but there has also been an increase in the incidence of residual MR ≤ 1, with these rates now reaching those observed in surgical interventions. In this view, the enhanced experience in treating MR, the advancements in ancillary therapy for HF, and the development of new-generation devices suggest a need for high-quality, comparative data concerning transcatheter and surgical treatment for DMR. In this regard, a number of clinical trials are investigating patients with lower-than-prohibitive risk for surgery (NCT05051033; NCT04198870; NCT03271762) [19,20]. It is noteworthy that the Percutaneous or Surgical Repair In Mitral Prolapse And Regurgitation for >60 Year-olds (PRIMARY) trial will be the only one to utilize both the Pascal and MitraClip devices for percutaneous MR repair.

In addition, due to the lack of clinical trials investigating this specific research question, it cannot be asserted that transcatheter repair leads to superior outcomes in comparison to surgery. Furthermore, the clinical benefit of TMVR is only maintained when compared to OMT alone. Further evidence is required to provide a robust comparison between TMVR and surgery, with the aim of clearly demonstrating that transcatheter therapies may represent an effective option for patients who are eligible for surgical treatment. Such evidence is expected to emerge from the results of ongoing trials.

Finally, we report the findings of the first network meta-analysis aiming at investigating the comparative efficacy of available treatment strategies for symptomatic patients with significant MR. Importantly, there are major differences in the amount of available evidence with respect to the efficacy and safety of each of the currently available treatment strategies. In addition, the predominant indirect nature of comparisons and the confounding role of underlying MR etiology should be taken into account when interpreting these results.

The present meta-analysis has revealed that the largest body of evidence currently available pertains to leaflet approximation procedures by means of the MitraClip device. The majority of trials included in the present meta-analysis (five out of seven) have incorporated this device as treatment. Accordingly, the MitraClip device ranked as the probable best treatment option in terms of the prevention of all-cause death. The only randomized trial regarding the use of the Pascal device is represented by the CLASP-IID trial, a comparison of the Pascal and MitraClip devices in patients diagnosed with predominantly DMR, concluding that the Pascal device is non-inferior. In the network meta-analysis, the Pascal device demonstrated a tendency towards superior outcomes in comparison to alternative treatment strategies, albeit without attaining statistical significance and exhibiting substantial heterogeneity. These data, therefore, should be interpreted with caution. Notwithstanding this fact, the two devices under scrutiny display distinct mechanical features that may give rise to divergent clinical outcomes. The Pascal device has nitinol paddles with a passive closure system and a central spacer whilst the MitraClip device has active grasping chromium–cobalt arms. It is hypothesized that the efficacy of MitraClip will be enhanced in the presence of calcified leaflets, and that post-procedural mitral gradients will be reduced with the Pascal device. Nonetheless, the results of observational studies [32,33,34] have failed to corroborate these theoretical advantages. Instead, these studies report largely comparable outcomes between the two devices. Further studies with the Pascal device are required in order to evaluate whether the favorable clinical outcomes observed following leaflet approximation procedures are related to a class effect, or whether there are significant differences between TEER devices.

Analogously, clinical evaluation of indirect annuloplasty by means of the Carillon device is limited to one RCT, characterized by a relatively limited sample size and insufficient power to evaluate hard clinical endpoints, comparing this treatment with OMT in patients with FMR. Indirect comparisons showed a trend towards better outcomes as compared to surgery or medical therapy, while the opposite was true when compared to TEER strategies. Due to the limited sample size, there was no evidence of statistical significance for any of these comparisons. However, our findings are in line with the findings of the CINCH registry [35]. Ongoing randomized trials, such as the EMPOWER trial (NCT03142152), will shed light on the clinical outcomes of patients undergoing treatment with the Carillon device.

Due to lack of available RCTs, clinical outcomes following transcatheter mitral valve implantation (TMVI), a more recent treatment option which has the potential to revolutionize the treatment of patients with MR, were not included in the present meta-analysis. However, results from feasibility studies [36] and registries [37] seem to indicate that TMVI is a valid alternative for patients not eligible for surgery or TEER. Future results from ongoing trials will clarify its role in the spectrum of MR treatments.

Some limitations of the present analysis should be acknowledged. First, this is a study-level meta-analysis and a patient-level meta-analysis remains the gold standard. Second, the majority of patients enrolled in the studies were at high surgical risk, reduced LVEF, and FMR, and the present results cannot be generalized to different patient populations. Third, the control arm in the pairwise meta-analysis is represented by both OMT and surgery, which may introduce a confounder; however, our results are supported by an adjunctive subgroup analysis by type of comparator. Fourth, the trials included in this analysis differed substantially in terms of study design, MR etiology, and type of comparator, resulting in high heterogeneity for the secondary outcomes. This, in turn, limited the robustness of our analysis for the summary estimates of hospitalization for heart failure, MR recurrence, re-interventions, and NYHA class. It must be acknowledged that the reliability of the results obtained from some of the secondary outcomes is questionable, due to the low number of events recorded in each group. Consequently, these results are statistically less credible, with large confidence intervals. In accordance with this aspect, the primary outcome constituted the exclusive object of the influence analysis. Fifth, direct comparisons between TMVR devices are extremely limited. Furthermore, comparative data, in particular for Pascal and Carillon devices, is mostly of an indirect nature. This underscores a fundamental constraint inherent network meta-analysis, which depends on indirect comparisons and the presumption of transitivity across studies. Such analyses are inherently subject to potential inconsistency between direct and indirect estimates, and their validity can be compromised by heterogeneity in study design, patient populations, and outcome definitions. We minimized these limitations through study selection and subgroup analyses, but results for secondary outcomes should be interpreted with caution.

## 5. Conclusions

In patients with symptomatic-relevant MR, primarily of functional origin, TMVR has been demonstrated to result in a reduction in mortality, hospitalization for heart failure, recurrent MR, and an improvement in functional status when compared to control therapy. However, due to the paucity of data, the potential benefits of TMVR cannot be extended to patients with degenerative MR. For such patients, surgery remains the gold standard treatment and TMVR should therefore be reserved for those with prohibitive surgical risk. Amongst the range of available transcatheter strategies, edge-to-edge repair with MitraClip has been potentially identified as the most effective procedure, due to the largest body of supporting evidence in comparison to alternative strategies. Further studies are required to evaluate the outcomes of newer-generation devices, particularly in the context of DMR in comparison to surgery, and to compare the currently available devices in order to ascertain potential differences in efficacy and to identify personalized treatment options for each individual patient with MR.

## Figures and Tables

**Table 1:** Main characteristic of the patients enrolled among the trials included in the study.

Trial	Patients, *n*	Age, Years	Male, %	Diabetes, %	STS Risk Score, %	COPD, %	DMR, %	FMR, %	LVEF, %	EROA, cm^2^	NYHA III–IV, %
CLASP IID	294	81	65	19	3.9	18	100	0	59	0.50	61
COAPT	614	72	64	37	8.15	23	0	100	31	0.41	60
EVEREST	279	67	64	9	N/R	15	73	27	60	0.58	68
MATTERHORN	208	71	60	26	2	17	0	100	43	0.22	86
MITRA FR	304	70	75	29	N/R	N/R	0	100	33	0.31	68
REDUCE FMR	120	70	73	30	N/R	N/R	0	100	34		54
RESHAPE HF2	505	70	80	35	5.3	14	0	100	32	0.23	75

STS, Society of Thoracic Surgeons; COPD, chronic obstructive pulmonary disease; DMR, degenerative mitral regurgitation; FMR, functional mitral regurgitation; LVEF, left ventricular ejection fraction; EROA, effective regurgitant orifice area; NYHA, New York Heart Association.

**Table 2:** Subgroup analysis for primary and secondary outcomes.

Variable	Subgroup	All-Cause Death	Pint	Re-Hospitalization for HF	Pint	Re-Intervention	Pint	MR 3+/4+	Pint	NYHA III or IV	Pint
Trial Size, patients	>300	0.80 [0.62–1.02]	0.87	0.60 [0.47–0.97]	0.92	0.39 [0.13; 1.16]	0.003	0.13 [0.09–0.2]	0.002	0.58 [0.48–0.70]	0.97
	≤300	0.75 [0.42–1.34]		0.69 [0.41–1.17]		5.07 [1.37; 18.79]		1.83 [0.36–9.19]		0.57 [0.22–1.47]	
Type of MR	FMR	0.77 [0.65–0.91]	0.65	0.67 [0.47–0.91]		0.61 [0.19; 1.97]	0.004	0.27 [0.08–0.86]	0.04	0.64 [0.49–0.85]	0.04
	DMR	0.59 [0.19–1.88]		Not estimable		9.10 [2.24; 36.89]		1.04 [0.63–1.71]		0.19 [0.06–0.57]	
Comparator	OMT	0.79 [0.64–0.97]	0.80	0.69 [0.50–0.95]	0.5	0.39 [0.13; 1.16]	0.003	0.13 [0.09–0.20]	0.002	0.58 [0.48–0.69]	0.91
	Surgery	0.72 [0.35–1.46]		0.43 [0.11–1.61]		5.07 [1.37; 18.79]		1.83 [0.36–9.19]		0.52 [0.08–3.45]	
Baseline % of NYHA III/IV	>65%	0.86 [0.68–1.09]	0.18	0.72 [0.43–1.21]	0.44	1.64 [0.13–20.71]	0.66	0.77 [0.14–4.30]	0.04	0.60 [0.27–1.31]	0.96
	≤65%	0.69 [0.55–0.87]		0.57 [0.43–0.76]		0.90 [0.41–1.96]		0.11 [0.06–0.20]		0.59 [0.45–0.76]	
Risk of Bias	High	0.71 [0.59–0.86]	0.08	0.54 [0.49–0.61]	0.002	1.02 [0.17; 6.12]	0.47	0.26 [0.06–1.08]	0.02	0.53 [0.39–0.74]	0.27
	Low or intermediate	1.01 [0.71–1.43]		0.91 [0.67–1.24]		2.50 [0.50; 12.59]		1.44 [0.11–1.73]		0.60 [0.44–0.84]	

DMR, degenerative mitral regurgitation; FMR, functional mitral regurgitation; HF, heart failure; MR, mitral regurgitation; NYHA, New York Heart Association; OMT, optimal medical therapy.

## Data Availability

The data supporting the study findings, including template data collection form, data extracted from included studies, and data used for all analyses, are available from the corresponding author upon reasonable request.

## Supplementary Materials

The following supporting information can be downloaded at https://www.mdpi.com/article/10.3390/jpm15080383/s1, Supplementary method: Statistical framework for pairwise meta-analyses. Figure S1: PRISMA 2020 flow diagram for searches of databases and registers; Figure S2: Risk of bias assessment for the included trials; Figure S3: Funnel plot for all-cause death; Figure S4: Network of treatment strategies for all-cause death; Figure S5: Forest plot from node-split model analysis for all-cause death; Figure S6: Comparison-adjusted funnel plot for all-cause death; Figure S7: Forest plot from network meta-analysis for re-hospitalization for heart failure; Figure S8: Forest plot from node-split model analysis for re-hospitalization for heart failure. Table S1: Search strategy; Table S2: Prisma statement; Table S3: Main characteristics of the included trials; Table S4: Influence analysis for all-cause death; [38,39,40,41].

## Abbreviations

The following abbreviations are used in this manuscript:

DMR	Degenerative mitral regurgitation
FMR	Functional mitral regurgitation
HF	Heart failure
MR	Mitral regurgitation
OMT	Optimal medical therapy
TMVR	Transcatheter mitral valve repair

## References

[1] Nkomo V.T., Gardin J.M., Skelton T.N., Gottdiener J.S., Scott C.G., Enriquez-Sarano M. (2006). Burden of valvular heart diseases: A population-based study. Lancet.

[2] Goel S.S., Bajaj N., Aggarwal B., Gupta S., Poddar K.L., Ige M., Bdair H., Anabtawi A., Rahim S., Whitlow P.L. (2014). Prevalence and outcomes of unoperated patients with severe symptomatic mitral regurgitation and heart failure. J. Am. Coll. Cardiol..

[3] Dziadzko V., Clavel M.-A., Dziadzko M., Medina-Inojosa J.R., Michelena H., Maalouf J., Nkomo V., Thapa P., Enriquez-Sarano M. (2018). Outcome and undertreatment of mitral regurgitation: A community cohort study. Lancet.

[4] Goldstein D., Moskowitz A.J., Gelijns A.C., Ailawadi G., Parides M.K., Perrault L.P., Hung J.W., Voisine P., Dagenais F., Gillinov A.M. (2016). Two-Year Outcomes of Surgical Treatment of Severe Ischemic Mitral Regurgitation. N. Engl. J. Med..

[5] Michler R.E., Smith P.K., Parides M.K., Ailawadi G., Thourani V., Moskowitz A.J., Acker M.A., Hung J.W., Chang H.L., Perrault L.P. (2016). Two-Year Outcomes of Surgical Treatment of Moderate Ischemic Mitral Regurgitation. N. Engl. J. Med..

[6] Goliasch G., Bartko P.E., Pavo N., Neuhold S., Wurm R., Mascherbauer J., Lang I.M., Strunk G., Hülsmann M. (2018). Refining the prognostic impact of functional mitral regurgitation in chronic heart failure. Eur. Heart J..

[7] Otto C.M., Nishimura R.A., Bonow R.O., Carabello B.A., Erwin J.P., Gentile F., Jneid H., Krieger E.V., Mack M., McLeod C. (2021). 2020 ACC/AHA Guideline for the Management of Patients with Valvular Heart Disease. Circulation.

[8] Vahanian A., Beyersdorf F., Praz F., Milojevic M., Baldus S., Bauersachs J., Capodanno D., Conradi L., De Bonis M., De Paulis R. (2022). 2021 ESC/EACTS Guidelines for the management of valvular heart disease. Eur. Heart J..

[9] Stone G.W., Lindenfeld J., Abraham W.T., Kar S., Lim D.S., Mishell J.M., Whisenant B., Grayburn P.A., Rinaldi M., Kapadia S.R. (2018). Transcatheter Mitral-Valve Repair in Patients with Heart Failure. N. Engl. J. Med..

[10] Obadia J.-F., Messika-Zeitoun D., Leurent G., Iung B., Bonnet G., Piriou N., Lefèvre T., Piot C., Rouleau F., Carrié D. (2018). Percutaneous Repair or Medical Treatment for Secondary Mitral Regurgitation. N. Engl. J. Med..

[11] Anker S.D., Friede T., von Bardeleben R.-S., Butler J., Khan M.-S., Diek M., Heinrich J., Geyer M., Placzek M., Ferrari R. (2024). Transcatheter Valve Repair in Heart Failure with Moderate to Severe Mitral Regurgitation. N. Engl. J. Med..

[12] Feldman T., Foster E., Glower D.D., Kar S., Rinaldi M.J., Fail P.S., Smalling R.W., Siegel R., Rose G.A., Engeron E. (2011). Percutaneous repair or surgery for mitral regurgitation. N. Engl. J. Med..

[13] Zahr F., Smith R.L., Gillam L.D., Chadderdon S., Makkar R., von Bardeleben R.S., Ruf T.F., Kipperman R.M., Rassi A.N., Szerlip M. (2023). One-Year Outcomes from the CLASP IID Randomized Trial for Degenerative Mitral Regurgitation. JACC Cardiovasc. Interv..

[14] Makkar R.R., Chikwe J., Chakravarty T., Chen Q., O’gAra P.T., Gillinov M., Mack M.J., Vekstein A., Patel D., Stebbins A.L. (2023). Transcatheter Mitral Valve Repair for Degenerative Mitral Regurgitation. JAMA.

[15] Anker M.S., Porthun J., Schulze P.C., Rassaf T., Landmesser U. (2024). Percutaneous Transcatheter Edge-To-Edge Repair for Functional Mitral Regurgitation in Heart Failure. J. Am. Coll. Cardiol..

[16] D’Amario D., Laborante R., Mennuni M., Adamo M., Metra M., Patti G. (2024). Efficacy and safety of trans-catheter repair devices for mitral regurgitation. Int. J. Cardiol..

[17] Higgins J.P.T., Altman D.G., Gøtzsche P.C., Jüni P., Moher D., Oxman A.D., Savović J., Schulz K.F., Weeks L., Sterne J.A.C. (2011). The Cochrane Collaboration’s tool for assessing risk of bias in randomised trials. BMJ.

[18] Page M.J., McKenzie J.E., Bossuyt P.M., Boutron I., Hoffmann T.C., Mulrow C.D., Shamseer L., Tetzlaff J.M., Akl E.A., Brennan S.E. (2021). The PRISMA 2020 statement: An updated guideline for reporting systematic reviews. BMJ.

[19] McCarthy P.M., Whisenant B., Asgar A.W., Ailawadi G., Hermiller J., Williams M., Morse A., Rinaldi M., Grayburn P., Thomas J.D. (2023). Percutaneous MitraClip Device or Surgical Mitral Valve Repair in Patients with Primary Mitral Regurgitation Who Are Candidates for Surgery: Design and Rationale of the REPAIR MR Trial. J. Am. Heart Assoc..

[20] Piriou N., Al Habash O., Donal E., Senage T., Le Tourneau T., Pattier S., Guyomarch B., Roussel J.-C., Trochu J.N., Vahanian A. (2019). The MITRA-HR study: Design and rationale of a randomised study of MitraClip transcatheter mitral valve repair in patients with severe primary mitral regurgitation eligible for high-risk surgery. EuroIntervention.

[21] Song C., Madhavan M.V., Lindenfeld J., Abraham W.T., Kar S., Lim D.S., Grayburn P.A., Kapadia S.R., Kotinkaduwa L.N., Mack M.J. (2022). Age-Related Outcomes After Transcatheter Mitral Valve Repair in Patients with Heart Failure: Analysis from COAPT. JACC Cardiovasc. Interv..

[22] Lindenfeld J., Abraham W.T., Grayburn P.A., Kar S., Asch F.M., Lim D.S., Nie H., Singhal P., Sundareswaran K.S., Weissman N.J. (2021). Association of Effective Regurgitation Orifice Area to Left Ventricular End-Diastolic Volume Ratio with Transcatheter Mitral Valve Repair Outcomes: A Secondary Analysis of the COAPT Trial. JAMA Cardiol..

[23] Shahim B., Cohen D.J., Asch F.M., Bax J., George I., Rück A., Ben-Yehuda O., Kar S., Lim D.S., Saxon J.T. (2024). Repeat Mitral Valve Interventions After Transcatheter Edge-to-Edge Repair: The COAPT Trial. Am. J. Cardiol..

[24] Witte K.K., Lipiecki J., Siminiak T., Meredith I.T., Malkin C.J., Goldberg S.L., Stark M.A., von Bardeleben R.S., Cremer P.C., Jaber W.A. (2019). The REDUCE FMR Trial: A Randomized Sham-Controlled Study of Percutaneous Mitral Annuloplasty in Functional Mitral Regurgitation. JACC Heart Fail..

[25] Baldus S., Doenst T., Pfister R., Gummert J., Kessler M., Boekstegers P., Lubos E., Schröder J., Thiele H., Walther T. (2024). Transcatheter Repair versus Mitral-Valve Surgery for Secondary Mitral Regurgitation. N. Engl. J. Med..

[26] Grayburn P.A., Sannino A., Packer M. (2019). Proportionate and Disproportionate Functional Mitral Regurgitation. JACC Cardiovasc. Imaging.

[27] von Bardeleben R.S., Mahoney P., Morse M.A., Price M.J., Denti P., Maisano F., Rogers J.H., Rinaldi M., De Marco F., Rollefson W. (2023). 1-Year Outcomes with Fourth-Generation Mitral Valve Transcatheter Edge-to-Edge Repair from the EXPAND G4 Study. JACC Cardiovasc. Interv..

[28] Kar S., von Bardeleben R.S., Rottbauer W., Mahoney P., Price M.J., Grasso C., Williams M., Lurz P., Ahmed M., Hausleiter J. (2023). Contemporary Outcomes Following Transcatheter Edge-to-Edge Repair: 1-Year Results from the EXPAND Study. JACC Cardiovasc. Interv..

[29] Okuno T., Izumo M., Shiokawa N., Kuwata S., Ishibashi Y., Sato Y., Koga M., Okuyama K., Suzuki N., Kida K. (2023). Newer versus Early Generation of the MitraClip for Primary Mitral Regurgitation: A Japanese Single-Center Experience. Rev. Cardiovasc. Med..

[30] Chakravarty T., Makar M., Patel D., Oakley L., Yoon S.H., Stegic J., Singh S., Skaf S., Nakamura M., Makkar R.R. (2020). Transcatheter Edge-to-Edge Mitral Valve Repair with the MitraClip G4 System. JACC Cardiovasc. Interv..

[31] Saji M., Yamamoto M., Kubo S., Asami M., Enta Y., Shirai S., Izumo M., Mizuno S., Watanabe Y., Amaki M. (2023). Short-Term Outcomes Following Transcatheter Edge-to-Edge Repair: Insights from the OCEAN-Mitral Registry. JACC Asia.

[32] Geis N.A., Schlegel P., Heckmann M.B., Katus H.A., Frey N., López P.C., Raake P.W. (2022). One-Year Results Following PASCAL-Based or MitraClip-Based Mitral Valve Transcatheter Edge-to-Edge Repair. ESC Heart Fail..

[33] Haschemi J., Haurand J.M., Bönner F., Kelm M., Westenfeld R., Horn P. (2022). PASCAL vs MitraClip for Mitral Valve Transcatheter Edge-to-Edge Repair: A Single-Center Real-World Experience. JACC Cardiovasc. Interv..

[34] Mauri V., Sugiura A., Spieker M., Iliadis C., Horn P., Öztürk C., Besler C., Riebisch M., Al-Hammadi O., Ruf T. (2022). Early Outcomes of 2 Mitral Valve Transcatheter Leaflet Approximation Devices: A Propensity Score-Matched Multicenter Comparison. JACC Cardiovasc. Interv..

[35] Yildiz M., Haude M., Sievert H., Fichtlscherer S., Lehmann R., Klein N., Witte K., Degen H., Pfeiffer D., Goldberg S.L. (2024). The CINCH-FMR postmarket registry: Real-world long-term outcomes with percutaneous mitral valve repair. Cardiovasc. Revasc. Med..

[36] Webb J.G., Murdoch D.J., Boone R.H., Moss R., Attinger-Toller A., Blanke P., Cheung A., Hensey M., Leipsic J., Ong K. (2019). Percutaneous Transcatheter Mitral Valve Replacement: First-in-Human Experience with a New Transseptal System. J. Am. Coll. Cardiol..

[37] Hell M.M., Wild M.G., Baldus S., Rudolph T., Treede H., Petronio A.S., Modine T., Andreas M., Coisne A., Duncan A. (2024). Transapical Mitral Valve Replacement: 1-Year Results of the Real-World Tendyne European Experience Registry. JACC Cardiovasc. Interv..

[38] Turner R.M., Bird S.M., Higgins J.P.T. (2013). The impact of study size on meta-analyses: Examination of underpowered studies in Cochrane reviews. PLoS ONE.

[39] Salanti G., Del Giovane C., Chaimani A., Caldwell D.M., Higgins J.P.T. (2014). Evaluating the quality of evidence from a network meta-analysis. PLoS ONE.

[40] Dias S., Welton N.J., Caldwell D.M., Ades A.E. (2010). Checking consistency in mixed treatment comparison meta-analysis. Stat. Med..

[41] Rucker G. (2012). Network meta-analysis, electrical networks and graph theory. Res. Synth. Methods.

