# Supplementary material for: Treatment Strategies for Patients with Mitral Regurgitation: A Meta-Analysis of Randomized Controlled Trials
Source: J Pers Med. 2025 Aug 16;15(8):383. doi: 10.3390/jpm15080383 (PMC12387918; doi:10.3390/jpm15080383)
Supplement: Supplementary file 1 [file jpm-15-00383-s001.zip › jpm-3741511-supplementary.pdf]

Supplementary material

This appendix has been provided by the authors to give readers additional information about their work.

Supplement to: Comparative effectiveness of treatment strategies in patients with mitral regurgitation: a network meta-analysis of randomized controlled trials

SUPPLEMENTARY METHOD 2

Statistical framework for pairwise meta-analyses. 2

SUPPLEMENTARY FIGURES 4

Figure S1. PRISMA 2020 flow diagram for searches of databases and registers. 4

Figure S2. Risk of bias assessment for the included trials. 5

Figure S3. Funnel plot for all-cause death. 6

Figure S4. Network of treatment strategies for all-cause death. 7

Figure S5. Forest plot from node-split model analysis for all-cause death. 8

Figure S6. Comparison-adjusted funnel plot for all-cause death. 9

Figure S7. Forest plot from network meta-analysis for re-hospitalization for heart failure. 10

Figure S8. Forest plot from node-split model analysis for re-hospitalization for heart failure. 11

SUPPLEMENTARY TABLES

Table S1. Search Strategy. 12

Table S2. Prisma statement. 13

Table S3. Main characteristics of the included trials. 16

Table S4. Influence analysis for all-cause death. 18

References of supplementary data 19

SUPPLEMENTARY METHOD

Statistical framework for pairwise meta-analyses.

For the pairwise meta-analyses, treatment effect was not assessed in trials in which no events were reported within-groups. The possibility of small study effects due to publication bias or other biases was examined for the primary outcome by means of visual inspection of funnel plots of the RRs of individual trials against their standard errors. A linear regression test for funnel plot asymmetry and influence analysis, in which meta-analysis estimates are computed omitting one study at a time, were performed for the primary outcome. Using a chi-square test for subgroup-by-treatment interaction, we tested whether the number of patients included in each trial (>300 versus ≤300), type of MR (functional versus degenerative), control treatment (surgery versus OMT), baseline functional status (> 65% of patients in NYHA III or IV class versus ≤65%) and risk of bias of each included trial (high versus low/intermediate) was associated with a modification of the treatment effect for the outcomes of interest. We calculated the power of our meta-analysis to detect a 25% relative risk difference for the primary outcome with TMVR conditional on the observed precision of the pooled estimate [38]. We set the 25% threshold as a benchmark because it corresponds to the mean assumption of superiority supporting the power usually used in clinical practice. Heterogeneity was assessed by the inconsistency factor (I^2), with <25% considered low, 25%-50% moderate, and >50% high [39]. Consistency between direct and indirect evidence was assessed by the node-splitting method (i.e., splitting the contributions to each comparison into direct and indirect evidence and assessing the contrast between the two components of evidence) [40]. Heterogeneity within study-to-study comparisons was further assessed by I^2 statistic. We provided a ranking of strategies for the main outcome based on P-scores according to Rücker et al. [41]. The P-score measures the average degree of certainty that a strategy or intervention is better than the competing ones. For instance, the P-score value is between 0 and 1: the higher the value, the greater the probability that a strategy or intervention is highly effective or safe, while a lower value shows that a strategy or intervention is ineffective.

The impact of small study effects and publication bias for the outcome all-cause death was examined by comparison-adjusted funnel plot and Egger's linear regression test.

SUPPLEMENTARY FIGURES

Figure S1. PRISMA 2020 flow diagram for searches of databases and registers.

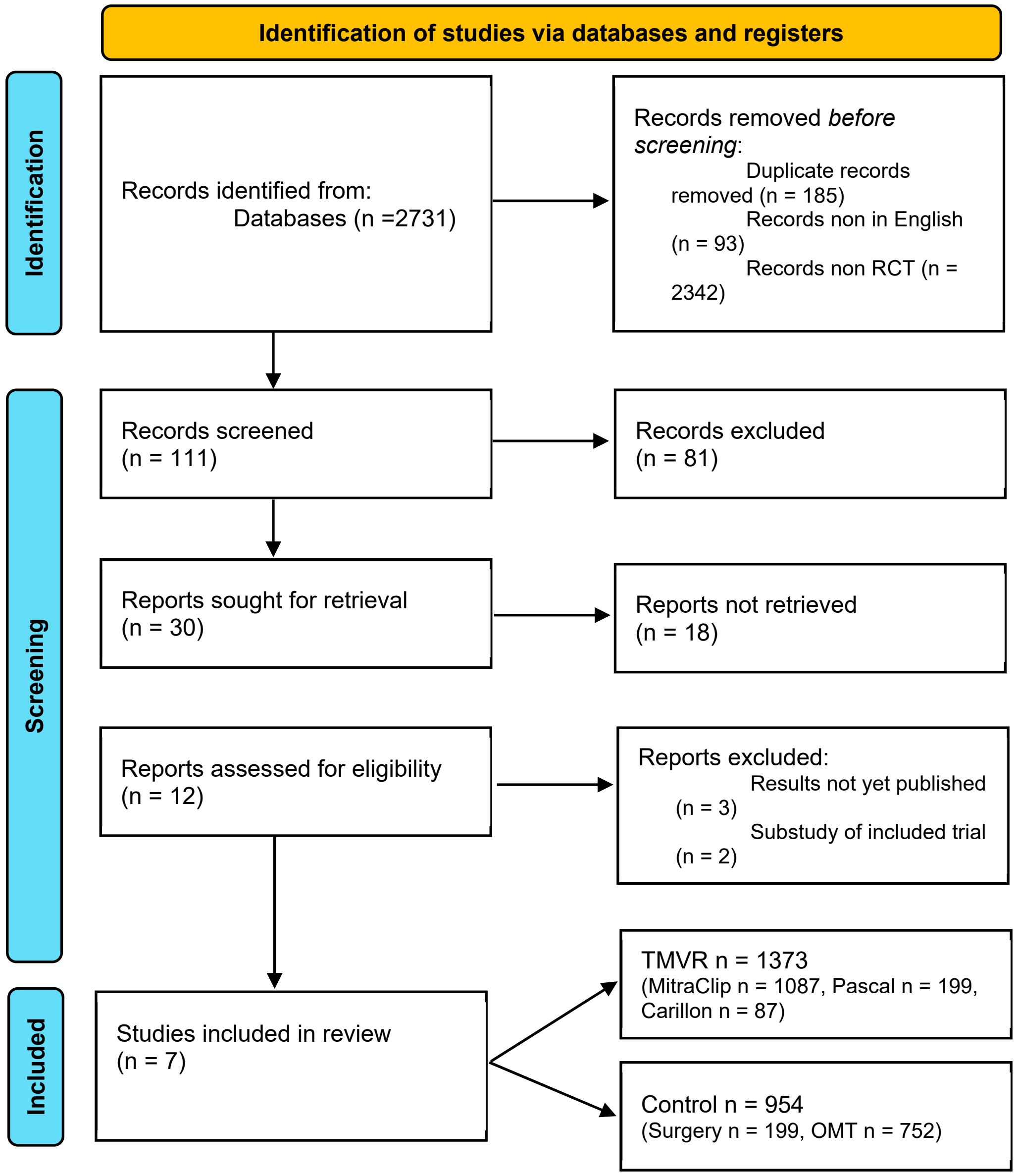

Figure S2. Risk of bias assessment for the included trials.

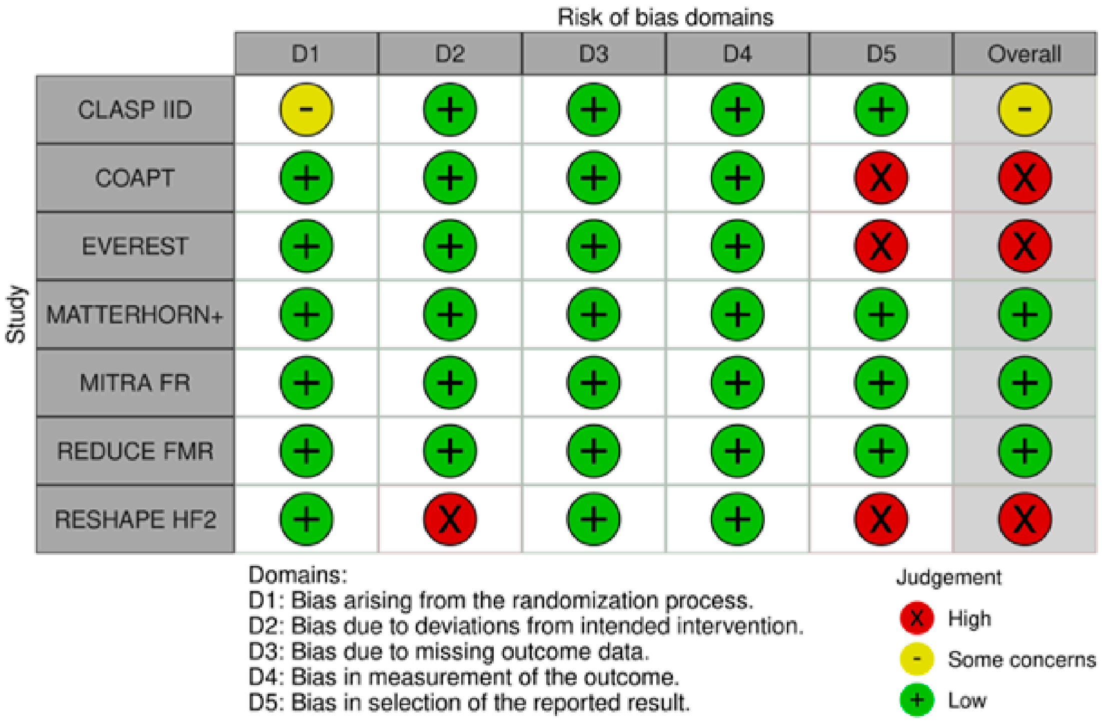

Figure S3. Funnel plot for all-cause death.

The distribution of the trials show substantial absence of publication bias. Trials are sorted basing on MR etiology, in FMR (red rhomboids) and DMR (black circles). DMR, degenerative mitral regurgitation; FMR, functional mitral regurgitation; MR, mitral regurgitation; RR, risk ratio; SE, standard error.

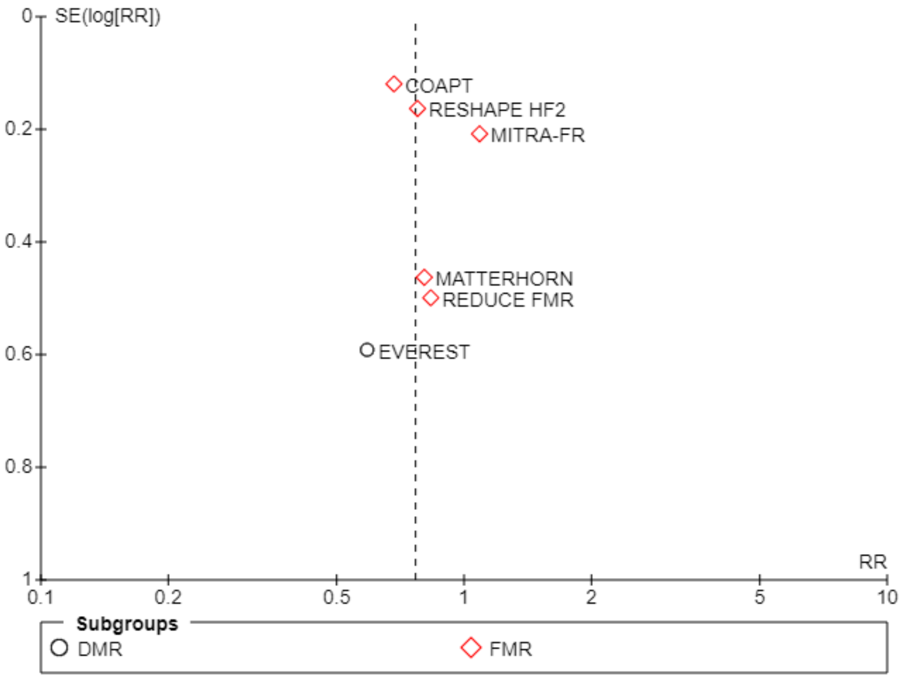

Figure S4. Network of treatment strategies for all-cause death.

The nodes in the graph layout correspond to the different treatment strategies and edges display the direct comparisons for all-cause death. The edge thickness is proportional to the number of comparisons. OMT, optimal medical therapy.

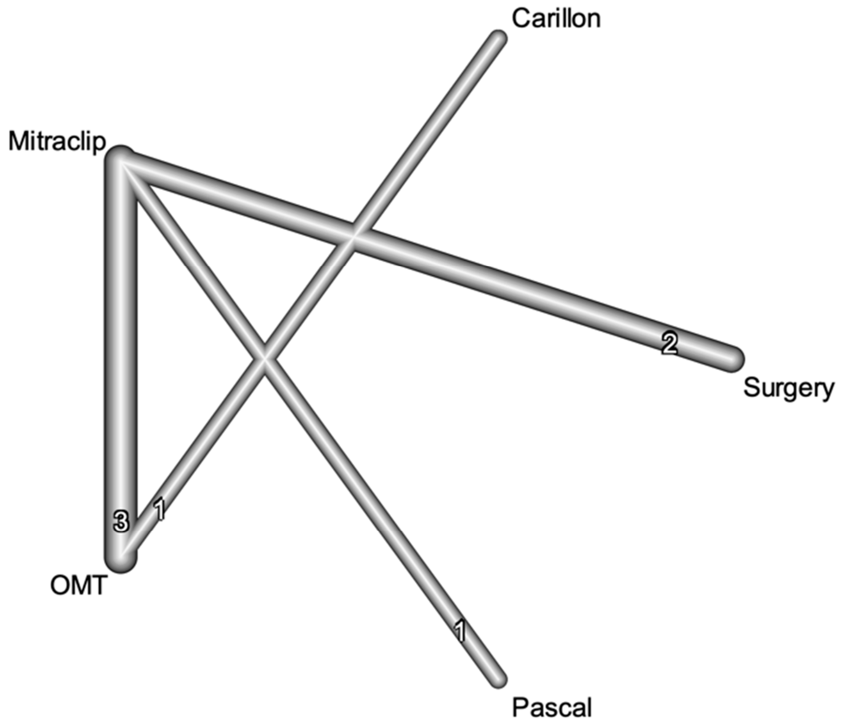

Figure S5. Forest plot from node-split model analysis for all-cause death.

The forest plots of pooled risk ratios and 95%CI for all cause death are derived by a node-splitting analysis of inconsistency between cumulated direct and indirect evidence. The number under the label “direct evidence” describes the proportion of direct evidence within the network estimate. RR, risk ratio; OMT, optimal medical therapy.

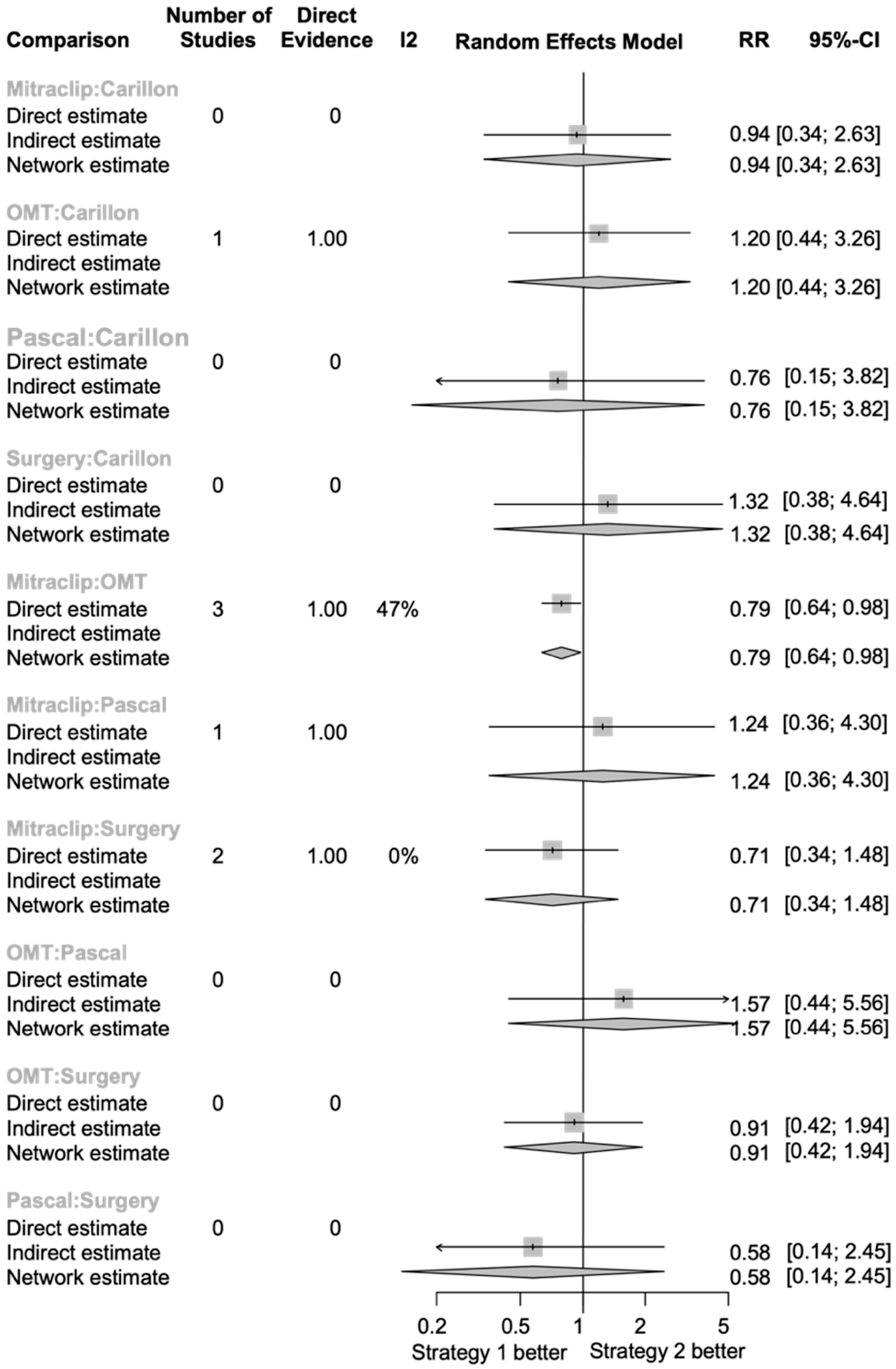

Figure S6. Comparison-adjusted funnel plot for all-cause death. The assessment of publication bias in the network meta-analysis for all-cause death was performed by defining an order for the hypothesized publication bias mechanism. OMT, optimal medical therapy.

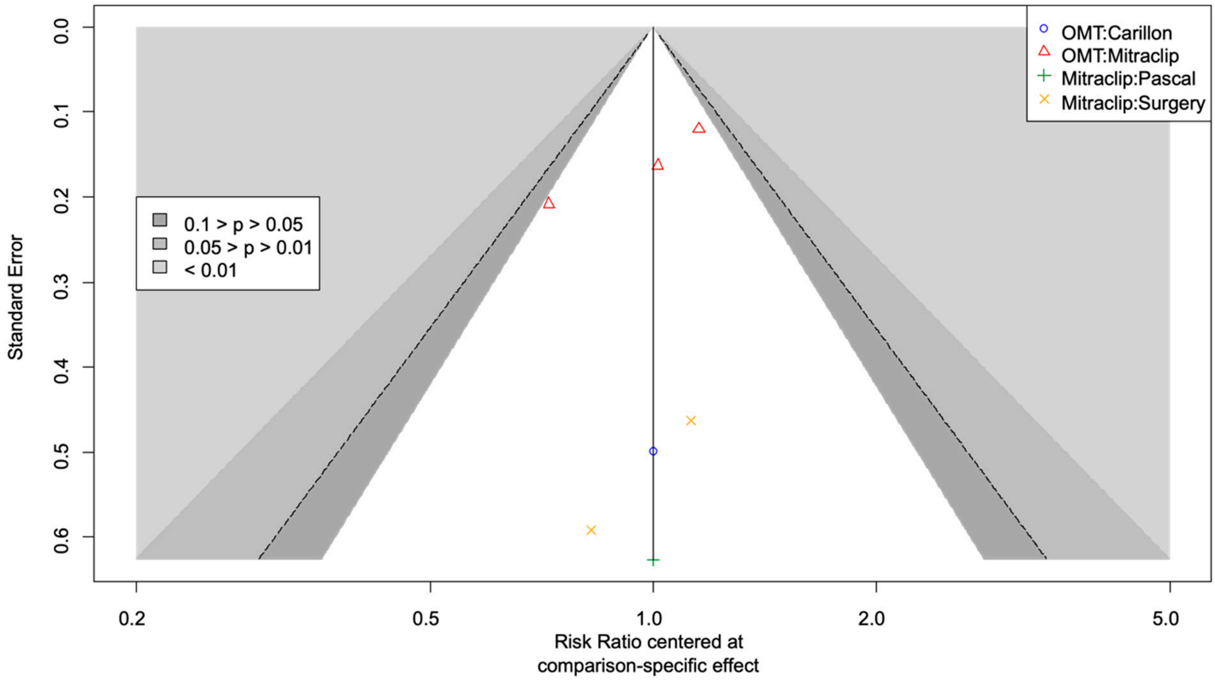

Figure S7. Forest plot from network meta-analysis for re-hospitalization for heart failure. The forest plots of pooled risk ratios and 95%CI for major bleeding are derived by network meta-analysis. RR, risk ratio; OMT, optimal medical therapy.

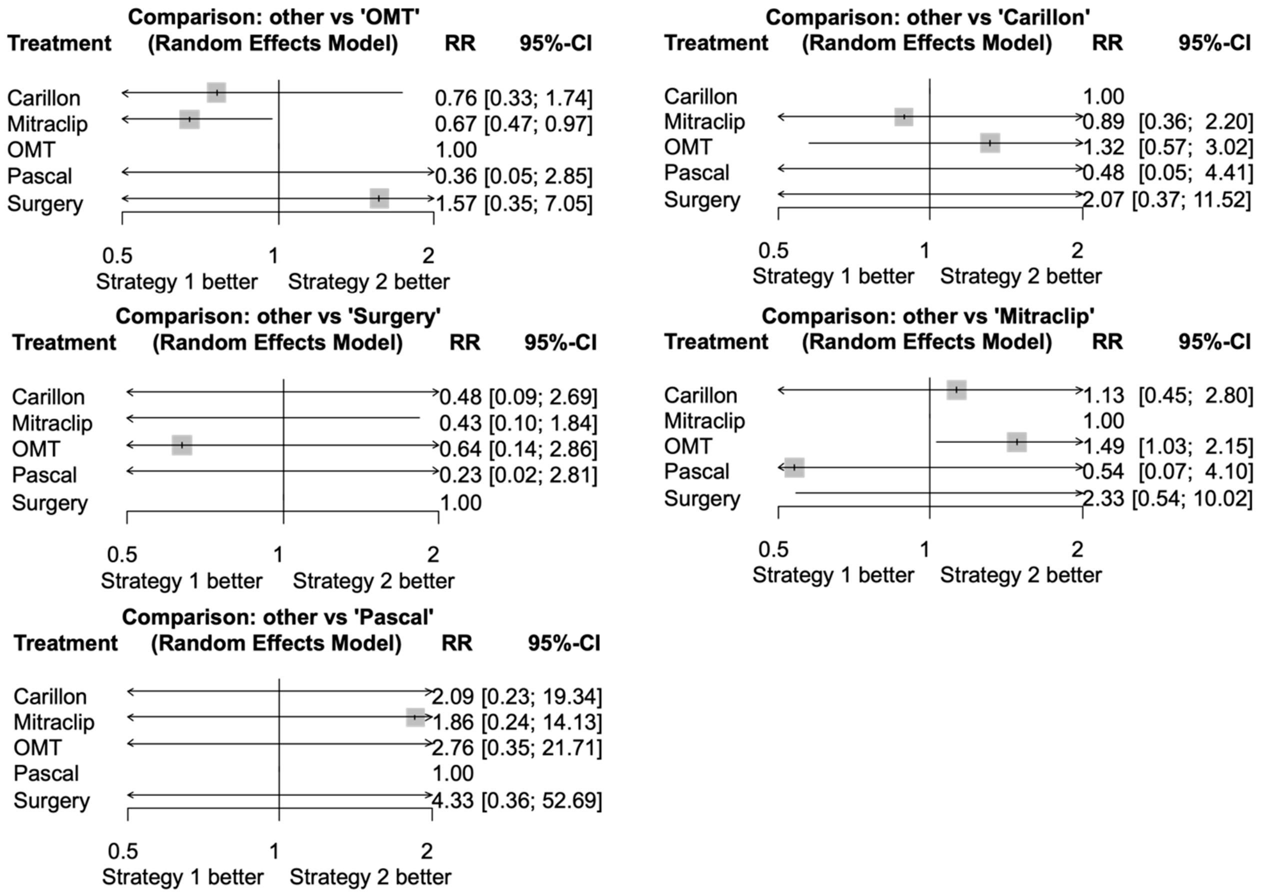

Figure S8. Forest plot from node-split model analysis for re-hospitalization for heart failure.

The forest plots of pooled risk ratios and 95%CI for heart failure hospitalization are derived by a node-splitting analysis of inconsistency between cumulated direct and indirect evidence. The number under the label “direct evidence” describes the proportion of direct evidence within the network estimate. RR, risk ratio; OMT, optimal medical therapy.

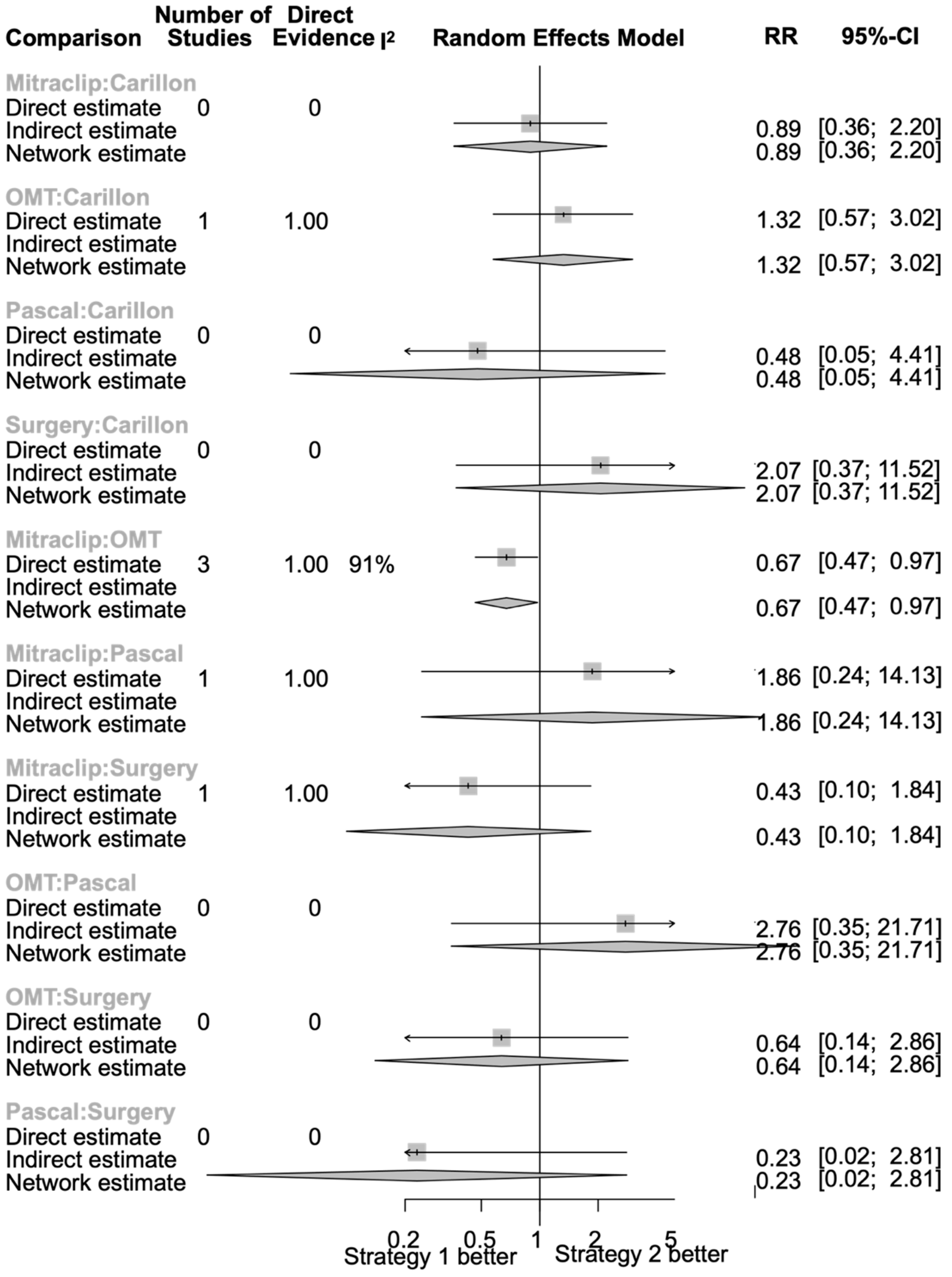

SUPPLEMENTARY TABLES

Table S1. Search Strategy.

EMBASE AND MEDLINE		
**Search line**	**Search Terms**	**No. citations**
**1**	**'structural heart disease'**:ab,ti AND ([embase]/lim OR [medline]/lim)	8132
**2**	**'mitral valve disease'**:ab,ti AND ([embase]/lim OR [medline]/lim)	5459
**3**	**mitral AND regurgitation:**ab,ti AND ([embase]/lim OR [medline]/lim)	2731
**4**	**'mitral valve regurgitation'**:ab,ti AND ([embase]/lim OR [medline]/lim)	2890
**5**	**mitral AND valve AND intervention**:ab,ti AND ([embase]/lim OR [medline]/lim)	7674
**6**	**'mitral valve repair'**:ab,ti AND ([embase]/lim OR [medline]/lim)	9639
**7**	**'transcatheter mitral valve repair'**:ab,ti AND ([embase]/lim OR [medline]/lim)	1002
**8**	**'percutaneous repair'**:ab,ti AND ([embase]/lim OR [medline]/lim)	680
**9**	**'edge to edge mitral valve repair'**:ab,ti AND ([embase]/lim OR [medline]/lim)	623
**10**	**'transcatheter edge to edge repair'**:ab,ti AND ([embase]/lim OR [medline]/lim)	947
**11**	**'mitral annuloplasty'**:ab,ti AND ([embase]/lim OR [medline]/lim)	1269
**12**	**mitraclip**:ab,ti AND ([embase]/lim OR [medline]/lim)	3539
**13**	**'pascal'**:ab,ti AND ([embase]/lim OR [medline]/lim)	2077
**14**	**'carillon'**:ab,ti AND ([embase]/lim OR [medline]/lim)	129
**15**	#3 AND #8	215
**16**	#3 AND #10	707
**15**	#3 AND #11	882
**16**	#7 OR #15 OR #16 OR #17	**2731**

Table S2. PRISMA Checklist.

Section and Topic	Item #	Checklist item	Location where item is reported
**TITLE**			
Title	1	Identify the report as a systematic review.	Title page
**ABSTRACT**			
Abstract	2	See the PRISMA 2020 for Abstracts checklist.	Title page
**INTRODUCTION**			
Rationale	3	Describe the rationale for the review in the context of existing knowledge.	Introduction
Objectives	4	Provide an explicit statement of the objective(s) or question(s) the review addresses.	Introduction
**METHODS**			
Eligibility criteria	5	Specify the inclusion and exclusion criteria for the review and how studies were grouped for the syntheses.	Methods, Search Strategy
Information sources	6	Specify all databases, registers, websites, organisations, reference lists and other sources searched or consulted to identify studies. Specify the date when each source was last searched or consulted.	Methods, Search Strategy
Search strategy	7	Present the full search strategies for all databases, registers and websites, including any filters and limits used.	Methods, supplementary Table 1 and supplementary Figure 1
Selection process	8	Specify the methods used to decide whether a study met the inclusion criteria of the review, including how many reviewers screened each record and each report retrieved, whether they worked independently, and if applicable, details of automation tools used in the process.	Methods search strategy
Data collection process	9	Specify the methods used to collect data from reports, including how many reviewers collected data from each report, whether they worked independently, any processes for obtaining or confirming data from study investigators, and if applicable, details of automation tools used in the process.	Methods, assessment of risk of bias
Data items	10a	List and define all outcomes for which data were sought. Specify whether all results that were compatible with each outcome domain in each study were sought (e.g. for all measures, time points, analyses), and if not, the methods used to decide which results to collect.	Methods search strategy and statistical analysis
	10b	List and define all other variables for which data were sought (e.g. participant and intervention characteristics, funding sources). Describe any assumptions made about any missing or unclear information.	Methods statistical analysis
Study risk of bias assessment	11	Specify the methods used to assess risk of bias in the included studies, including details of the tool(s) used, how many reviewers assessed each study and whether they worked independently, and if applicable, details of automation tools used in the process.	Methods, Assessment of risk of bias
Effect measures	12	Specify for each outcome the effect measure(s) (e.g. risk ratio, mean difference) used in the synthesis or presentation of results.	Methods, statistical analysis

Section and Topic	Item #	Checklist item	Location where item is reported
Synthesis methods	13a	Describe the processes used to decide which studies were eligible for each synthesis (e.g. tabulating the study intervention characteristics and comparing against the planned groups for each synthesis (item #5)).	Supplementary materials
	13b	Describe any methods required to prepare the data for presentation or synthesis, such as handling of missing summary statistics, or data conversions.	Methods, statistical analysis
	13c	Describe any methods used to tabulate or visually display results of individual studies and syntheses.	Methods, statistical analysis
	13d	Describe any methods used to synthesize results and provide a rationale for the choice(s). If meta-analysis was performed, describe the model(s), method(s) to identify the presence and extent of statistical heterogeneity, and software package(s) used.	Methods, statistical analysis
	13e	Describe any methods used to explore possible causes of heterogeneity among study results (e.g. subgroup analysis, meta-regression).	Methods, statistical analysis
	13f	Describe any sensitivity analyses conducted to assess robustness of the synthesized results.	Methods, statistical analysis
Reporting bias assessment	14	Describe any methods used to assess risk of bias due to missing results in a synthesis (arising from reporting biases).	Methods, Assessment of risk of bias
Certainty assessment	15	Describe any methods used to assess certainty (or confidence) in the body of evidence for an outcome.	Methods, statistical analysis
**RESULTS**			
Study selection	16a	Describe the results of the search and selection process, from the number of records identified in the search to the number of studies included in the review, ideally using a flow diagram.	Results, Eligible and included studies
	16b	Cite studies that might appear to meet the inclusion criteria, but which were excluded, and explain why they were excluded.	Results, Eligible and included studies
Study characteristics	17	Cite each included study and present its characteristics.	Results, Table 1 and Supplementary Table 3
Risk of bias in studies	18	Present assessments of risk of bias for each included study.	Supplementary Figure 2
Results of individual studies	19	For all outcomes, present, for each study: (a) summary statistics for each group (where appropriate) and (b) an effect estimate and its precision (e.g. confidence/credible interval), ideally using structured tables or plots.	Figures 1-3, supplementary material
Results of syntheses	20a	For each synthesis, briefly summarise the characteristics and risk of bias among contributing studies.	Results, Supplementary figures 2 and 3
	20b	Present results of all statistical syntheses conducted. If meta-analysis was done, present for each the summary estimate and its precision	Results primary

Section and Topic	Item #	Checklist item	Location where item is reported
		(e.g. confidence/credible interval) and measures of statistical heterogeneity. If comparing groups, describe the direction of the effect.	and secondary outcomes
	20c	Present results of all investigations of possible causes of heterogeneity among study results.	Results, all
	20d	Present results of all sensitivity analyses conducted to assess the robustness of the synthesized results.	Results, all
Reporting biases	21	Present assessments of risk of bias due to missing results (arising from reporting biases) for each synthesis assessed.	Supplementary material
Certainty of evidence	22	Present assessments of certainty (or confidence) in the body of evidence for each outcome assessed.	Supplementary figures 3-8
**DISCUSSION**			
Discussion	23a	Provide a general interpretation of the results in the context of other evidence.	Discussion
	23b	Discuss any limitations of the evidence included in the review.	Limitations
	23c	Discuss any limitations of the review processes used.	Limitations
	23d	Discuss implications of the results for practice, policy, and future research.	Conclusion
**OTHER INFORMATION**			
Registration and protocol	24a	Provide registration information for the review, including register name and registration number, or state that the review was not registered.	Methods section, final part
	24b	Indicate where the review protocol can be accessed, or state that a protocol was not prepared.	Methods section, final part
	24c	Describe and explain any amendments to information provided at registration or in the protocol.	NA
Support	25	Describe sources of financial or non-financial support for the review, and the role of the funders or sponsors in the review.	Funding
Competing interests	26	Declare any competing interests of review authors.	Conflict of interest
Availability of data, code and other materials	27	Report which of the following are publicly available and where they can be found: template data collection forms; data extracted from included studies; data used for all analyses; analytic code; any other materials used in the review.	Data Availability Statement

Table S3. Main characteristics of the included trials.

Trial	Design	Primary endpoint	Secondary endpoint	FU, months
**CLASP IID**	Pascal vs Mitraclip	MR reduction.	Adverse events; increase in 6MWD; improvement in QoL (KCCQ, SF-36, EQ-5D-5L questionnaire).	6
**COAPT**	Mitraclip vs OMT	(i) All hospitalization for HF; (ii) freedom from device-related complications (single leaflet device attachment, device embolization, endocarditis requiring surgery, mitral stenosis requiring surgery, LVAD implant; HTx, device related complication requiring non-elective cardiovascular surgery).	MR ≤2+; all-cause mortality; improvement in QoL (measured by KCCQ), increase in 6MWD, NYHA Class, change in LVEDV.	24
**EVEREST II**	Mitraclip vs Surgery	(i) Composite of death, MI, reintervention, non-elective cardiovascular surgery, stroke, renal failure, deep wound infection, ventilation > 48h, gastrointestinal complication requiring surgery, new onset AF, septicemia, transfusion ≥ 2 units of blood; (ii) Freedom from surgery for valve dysfunction, death, MR >2+.	Changes in LV dimensions and volumes; NYHA Class, improvement in QoL (SF-36 questionnaire).	12
**MATTERHORN**	Mitraclip vs Surgery	(i) Composite of death, HF hospitalization, reintervention, assist device implantation, stroke (ii) composite of death, MI, major bleeding, stroke or TIA, reintervention or non-elective cardiovascular surgery, renal replacement therapy, deep wound infection, ventilation > 48h, gastrointestinal complication requiring surgery, new-onset AF, septicemia, endocarditis.	MR 3+ or 4+ recurrence; increase in 6MWD; NYHA Class; improvement in QoL (Minnesota Living with Heart Failure Questionnaire).	12
**MITRA FR**	Mitraclip vs OMT	Composite of death or unplanned hospitalization for HF.	All-cause mortality; HF hospitalization; Freedom from death, stroke, MI, HF hospitalization; change in LV function and dimensions; MR grade; increase in 6MWD;	12

			NYHA Class; improvement in QoL (5–Dimensions scale); BNP.	
**REDUCE FMR**	Carillon vs OMT	Change in mitral regurgitant volume.	Adverse events (death, MI, device embolization, vessel erosion, cardiac perforation, cardiac surgery, PCI associated with device failure, HF hospitalizations); LV dimensions; NYHA Class; improvement in QoL (KCCQ).	12
RESHAPE HF2	Mitraclip vs OMT	(i) composite of HF hospitalizations and cardiovascular death; (ii) HF hospitalizations; (iii) change in KCCQ score	MR ≥ 2+; increase in 6MWD; all-cause death; recurrent HF hospitalizations; NYHA Class.	24

MR, Mitral regurgitation; 6MWD, 6 minutes walking distance; QoL, quality of life; KCCQ, Kansas City Cardiomyopathy Questionnaire; SF-36, Short Form (36) Health Survey; EQ-5D-5L, EuroQol 5-Dimension 5-Level Questionnaire; OMT, optimal medical therapy; HF, heart failure; LVAD, left ventricular assist device; HTx, heart transplantation; LVEDV, left ventricular end-diastolic volume; NYHA, New York Heart Association; MI, myocardial infarction; AF, atrial fibrillation; LV, left ventricular; BNP, brain natriuretic peptide; MAE, major adverse events; PCI, percutaneous coronary intervention; TIA; transient ischemic attack.

Table S4. Influence analysis for all-cause death.

Trial ommitted	RR [95% CI]	P value	I^2%
**COAPT**	0.82 [0.61; 1.10]	0.19	0
**EVEREST**	0.72 [0.56; 0.92]	0.01	11
**MATTERHORN**	0.70 [0.52; 0.94]	0.02	29
**MITRA FR**	0.63 [0.49; 0.80]	0.0002	0
**REDUCE FMR**	0.70 [0.52; 0.94]	0.02	29
**RESHAPE HF2**	0.71 [0.49; 1.02]	0.06	30
